# 3D Cell Models in Radiobiology: Improving the Predictive Value of In Vitro Research

**DOI:** 10.3390/ijms241310620

**Published:** 2023-06-25

**Authors:** Francesca Antonelli

**Affiliations:** Laboratory of Biomedical Technologies, Division of Health Protection Technologies, Agenzia Nazionale per le Nuove Tecnologie, l’Energia e lo Sviluppo Economico Sostenibile (ENEA), 00123 Rome, Italy; francesca.antonelli@enea.it

**Keywords:** 3D cell models, Linear-quadratic model, dose–response curves, 3D bioprinting, organoids, organ-on-a-chip, radiobiology

## Abstract

Cancer is intrinsically complex, comprising both heterogeneous cellular composition and extracellular matrix. In vitro cancer research models have been widely used in the past to model and study cancer. Although two-dimensional (2D) cell culture models have traditionally been used for cancer research, they have many limitations, such as the disturbance of interactions between cellular and extracellular environments and changes in cell morphology, polarity, division mechanism, differentiation and cell motion. Moreover, 2D cell models are usually monotypic. This implies that 2D tumor models are ineffective at accurately recapitulating complex aspects of tumor cell growth, as well as their radiation responses. Over the past decade there has been significant uptake of three-dimensional (3D) in vitro models by cancer researchers, highlighting a complementary model for studies of radiation effects on tumors, especially in conjunction with chemotherapy. The introduction of 3D cell culture approaches aims to model in vivo tissue interactions with radiation by positioning itself halfway between 2D cell and animal models, and thus opening up new possibilities in the study of radiation response mechanisms of healthy and tumor tissues.

## 1. Introduction

Two-dimensional (2D) cell cultures have historically been used for radiobiological studies and for modelling interactions between radiation and tissues, and so many of the dogmas of radiobiology are based on cellular and molecular responses of cells grown attached to a plastic surface [1,2,3]. In particular, the “gold standards” used to assess sensitivity towards radiotherapy are represented by the “clonogenic assay”, a test used to measure reproductive cell survival in vitro [4,5], and DNA damage evaluation in established cell lines [6,7]. Although these approaches are well-accepted, contributing significantly to the understanding of the mechanisms underlying cellular response to radiation, 2D systems depict an oversimplified representation of reality. For example, 2D cell cultures are usually monotypic, being made up of only a single cell type. In these models, the paracrine signaling between cells of different types is completely abolished. The tumor microenvironment, made up of both malignant cells and nonmalignant cellular and non-cellular components, can heavily condition the disease initiation, progression and treatment response of the tumor, since malignant cells and stromal components reciprocally communicate. Cancer-associated fibroblasts, as well as other tumor-associated cells, including endothelial cells and adipocytes [8], can highly increase tumor radioresistance through pro-survival factor secretion, immunomodulatory signals and contact-mediated signaling [9]. Under 2D growth conditions, cell–cell interactions, as well as extracellular matrix interactions, which are important for proliferation, differentiation and normal cell function in vivo, are altered [10,11,12]. For anchorage-dependent cells, adhesive interactions with the ECM and neighboring cells are crucial to define shape and space organization, gene expression, proliferation rate, response to stimuli and drug metabolism, all important factors able to regulate the tight coupling between cell structure, signaling and function [12,13,14]. Moreover, expression of cell integrins, known to be well-documented mechanosensors, is deeply regulated by the mechanical properties of the ECM, which therefore plays a key role in cellular function and behavior [15,16].

Recent progress in cell biology, microfabrication techniques and tissue engineering has led to significant advancements in the field of 3D cell culture technologies. These advancements encompass various approaches including organoids, scaffolds, hydrogels, organs-on-chips and 3D bioprinting, each offering unique advantages and disadvantages. Despite their distinct principles and protocols, these 3D culture methods aim to recapitulate the morphological, functional and microenvironmental characteristics of human tissues and organs. Three-dimensional cell cultures have been proven to mimic key factors of tissues in a more representative way, revealing themselves to have the potential to change the way in which tumors and tumor treatments are studied and modeled [10,11,12,17,18]. Growing evidence reported in the literature shows that increasing the dimensionality of ECM around cells, and switching from 2D to 3D models, can significantly impact cell proliferation, differentiation, cell-survival and, above all, the response of cells to external stimuli and insults [9,19,20,21,22].

Radiation therapy (RT) is a powerful anti-cancer treatment used to treat up to 50–60% of cancer patients, often in combination with systemic agents [23]. Different types of cancer are characterized by different tumor microenvironments that greatly affect the effectiveness of chemotherapy and radiotherapy treatments.

Over the years, the discrepancy between the expected results and those obtained after radiotherapy is becoming increasingly evident, and this can be partially related to the increased radioresistance shown by cells grown in 3D compared to those grown in a 2D microenvironment [24].

Tissue engineering is advancing as a promising approach to produce biomimetic 3D models able to recapitulate structural, biophysical, biochemical and biomechanical features of tumor tissues, and can be used for the study of tumor response to radiotherapy and further clinical application.

However, the application of 3D models in radiation response studies is currently an understudied area of research. Horvath et al. reported that the common and persistent failures to translate promising preclinical drug candidates into clinical success highlight the limited effectiveness of the disease models currently used in drug discovery [25]. An apparent reluctance to explore and adopt alternative cell- and tissue-based model systems, coupled with a detachment from clinical practice during assay validation, contributes to ineffective translational research.

## 2. The Linear–Quadratic Model and the Implications of Cellular Radioresistance in a 3D Environment

The clinical treatment of tumors usually includes surgery, radiotherapy and chemotherapy, depending on the type of cancer and on its staging and progression. In developing new radiation treatments, in vitro and in vivo studies are essential before moving on to clinic. The goal of radiotherapy is to eliminate highly proliferative cancer cells, mainly damaging DNA while sparing the healthy tissues surrounding the tumor site. The relationship between the probability of tumor control and the likelihood of normal tissue damage is based on differences in the DNA repair efficiency between cancer and normal cells. Historically, the prediction of the radiobiological response has been based on the use of radiobiological models and, among these, the linear–quadratic model (LQ) has been best validated by experimental and clinical data [26]. This model is based on a “cell survival” model of radiation response and describes the survival probability of a cell following exposure to a single dose of radiation, *SF*(*D*), as:

$$SF\left(D\right)=e^{-\alpha D-\beta D^{2}}$$

where α and β are the parameters describing the cell’s radiosensitivity, and D is the dose to which it is exposed. The α and β parameters describe the linear and quadratic portion of the survival curves, respectively, and their ratios differ widely across and even within some tumor types, giving fundamental indications on cellular sensitivity to radiation and on the advantages of providing small doses of radiation over a protracted treatment time (fractionated radiotherapy) [26,27]. Several modifications toward more sophisticated models have been developed over time [27], but all are based on experimental data obtained from 2D cell cultures. However, 2D cell models lack the ability to account for the effects of cellular growth in a three-dimensional environment, as occurs in reality, and the effects of the extracellular environment. Therefore, although the main role of the LQ model in radiotherapy is recognized, questions remain about its applicability, since it is increasingly evident that the tissue response to radiation is modulated both by genetic factors and extrinsic factors such as the tumor microenvironment (TME) [9,28,29,30]. Growing evidence indicates that TME is a key factor in tumor response to radiation, shifting from a cancer cells-centered view to a tumor-microenvironment-centered view. The cellular stromal component of many cancers includes activated cancer-associated fibroblasts (CAF), endothelial cells, immune cells, adipose cells and normal tissue cells [9,31,32]. The recapitulating of TME is an important challenge for understanding the mechanisms underlying the biological responses to radiation in a controlled and reproducible in vitro system. Much evidence has accumulated in recent years indicating a difference in radiosensitivity between cells growing in a 2D or 3D environment, and the majority of these studies have shown an increase in radioresistance in 3D cultures [15,24,33,34]. This is reflected in differences, which can be very significant, between survival curves obtained from 2D or 3D models [35,36]. For this reason, the exclusive use of 2D cellular models for the definition of survival curves and the application of the LQ model to radiotherapy plans could lead to an underestimation of the radiation dose necessary to eradicate the tumor and to an incorrect assessment of the risk–benefit ratio.

To the author’s current knowledge, the LQ model represents the best approximation that can describe the biological processes that determine the response to radiation. Ignoring the effect that the cellular microenvironment can have on the mechanisms underlying this response can contribute to vulnerability and a possible reduction in the effectiveness of radiotherapy plans.

## 3. Radioresistance Mechanisms in 3D Cell Models

### 3.1. Increased Stemness

Tumors are highly heterogeneous structures consisting of both many differentiated cancer cells and a small component of cancer stem cells (CSCs, <0.001% of tumor cells) [18]. There is much evidence to support the notion that CSCs contribute significantly to self-renewal activities leading to tumor growth, maintenance and metastasis and, above all, to chemo- and radioresistance [37,38,39]. Indeed, tumor treatment with radiation or therapeutic compounds can lead to enrichment of the stem cell population due to their ability to survive and proliferate after therapies [40]. Many recent studies report that CSCs possess a capacity for highly efficient DNA damage repair [37,41,42], the ability to reorganize the ECM [40,43] and interact with the TME [44,45], all factors implicated in resistance to anticancer therapies.

In recent years, it has become clear that the stem properties of cells strongly depend on the environment in which they grow. Suzuka et al. demonstrated that any of six human cancer cell lines or brain cancer cells resected from patients with glioblastoma (GBM) rapidly reprogrammed themselves into CSCs when seeded in a double-PEG hydrogel network [46]. Casciati et al. reported that a GBM cell line (U87), commonly used for in vitro studies, showed a modulation of the expression pattern of stemness/differentiation genes when cells were grown as neurospheres, with a switch toward a stem phenotype [47]. Xue et al. showed that an adenocarcinomic human alveolar basal epithelial cell line (A549 cell line) upregulated the gene and protein expression of the stem cell reprogramming factors (OCT4, SOX2, NANOG, LIN28 and miR-302a) when cultured in a Matrigel basement membrane matrix [35].

Many other works exist reporting the upregulation of stemness gene expression when cells are grown in a three-dimensional context, in which the type of 3D culture is not as important as the growth itself in a 3D environment [48,49,50,51].

### 3.2. Radioresistant DNA Damage in 3D Models

Radiotherapy exerts its effect mainly by inducing DNA damage, which leads to the activation of the cell DNA repair machinery. The most damaging effect of radiation is considered to be the induction of double-strand DNA breaks (DSB), which poses a potent threat to genomic integrity since unrepaired DNA damage activates the DNA damage response (DDR) signaling, which, in turn, induces apoptosis, senescence or mitotic catastrophe leading to the loss of the cell’s reproductive capacity [52,53,54,55,56]. In theory, every tumor cell can be destroyed with radiotherapy using a high enough dose. However, having to take into account the risks for the surrounding healthy tissue, the radiation doses used in radiotherapy treatments are often insufficient to completely eliminate the CSCs, which therefore represent the key factor of locoregional or distant recurrence. CD133^+^ cancer cells, a stem cell population capable of driving tumor growth [57], frequently undergo enrichment after radiotherapy treatment due to the greater efficiency of stem cells in DNA repair [58,59,60]. This enrichment seems to be partially due to the CSCs ability to activate the checkpoint kinase 1 (CHK1) and CHK2 through enhanced activation of ataxia-telangectasia-mutated (ATM) and ATM- and Rad3-Related (ATR) proteins [61,62,63]. Moreover, most studies suggest that the accurate homologous recombination (HR) can be of outstanding importance for the DNA DSB repair pathways of CSCs, whereas, for the more error-prone non-homologous end joining (NHEJ), only ATM-mediated effects are observed [64,65]. However, the mechanisms underlying the DDR in CSCs are still not clear. A number of studies have shown no difference or even lower DDR in CDCs [64,66], also considering intra-tumoral heterogeneity and that CSCs can represent a transient cell population [66].

Our current knowledge of the repair mechanisms of cancer cells is mainly based on the response of 2D cell cultures irradiated with low and high linear energy transfer (LET) radiations. Moreover, very limited data regarding the DNA repair process in 3D models are available. Akolawala et al. used biomimetic scaffolds to create in vitro replicas of the in vivo GBM microenvironment. The authors reported that the GBM cells cultured on 3D microenvironments qualitatively and quantitatively showed less H2AX foci, indicative of DNA DSB, than the cells cultured in 2D conditions, after irradiation with 2 and 8 Gy of protons [34]. A few authors reported similar observations about H2AX foci, highlighting differences in the DSB induction and repair in 2D or 3D growing conditions [67,68]. Moreover, as tumor radioresistance is also dependent on the radiation-induced changes of stem-like cell content, and given the higher efficiency of CSCs in DNA repair, it is essential to understand the link between stemness, cellular response to DNA damage and radiation in a three-dimensional environment capable of modulating the expression of stemness genes.

The effectiveness of radiotherapy plans is strongly based on the cellular DDR to radiation, which for a long time has been studied mainly on 2D culture systems. It is important to note that the first few available studies on DNA damage induction and repair in 3D tissue models suggested different mechanisms of response to radiation, but an apparent reluctance to explore and adopt alternative cell- and tissue-based model systems remains. The next challenge for radiobiological studies should be to recapitulate the complexity of the tumor environment and better understand how cellular growth in a three-dimensional environment influences the cellular response to DNA damage, to improve the efficacy of the radiotherapy plans.

### 3.3. The Radioprotective Role of the Tumor Microenvironment

**Figure 1:**
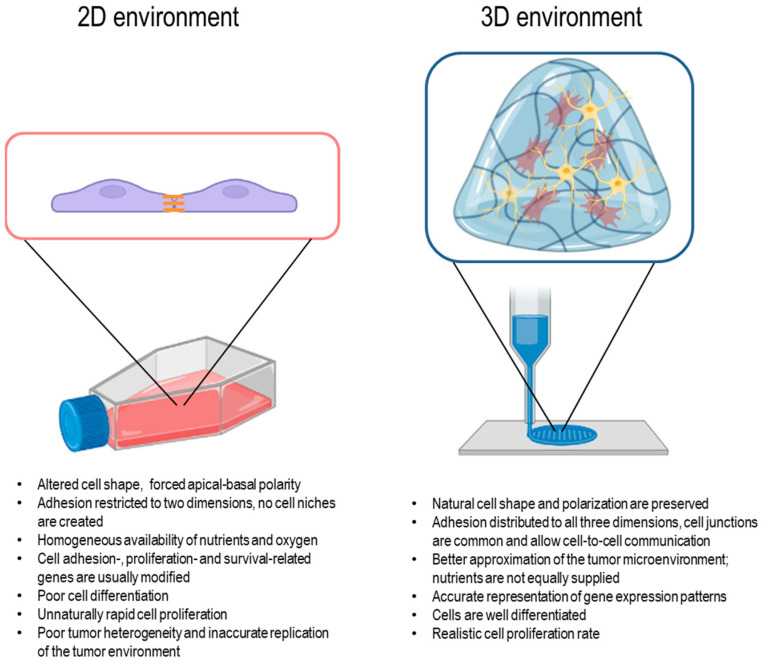
Schematic representation of the main differences between 2D and 3D cell cultures. Created with BioRender.com and adapted from free BioRender templates (2020).

The microenvironment of tumor cells has a profound impact on their physiology and radiation response. Traditionally, two-dimensional cell cultures are unable to provide and mimic an environment as complex as a real tissue. In particular, cells cultured on a flat and rigid support lack dimensionality, provide a highly polarized rather than homogeneous mechanical environment and are unable to maintain local concentration heterogeneities. Moreover, 2D cell cultures show a low ability to differentiate and lack the complex 3D architecture of the TME (Figure 1). For this reason, these models cannot recapitulate the intracellular, intercellular and cell-ECM interactions at the base of tumor growth, dissemination and response to radio- and chemotherapy.

TME exerts its radioprotective effects at different levels and is fundamental in the modulation of tumor radioresistance. Furthermore, it would be important to note that there are reports that radiotherapy can cause numerous changes in stromal cells which may be a contributing cause of undesirable effects such as tumor growth, invasion and resistance to treatments [69]. The TME can act at different levels in the modulation of cell response to radiation, including (a) ECM stiffness and mechanical force signals; (b) tumor stroma cell composition.

#### 3.3.1. ECM Stiffness and Mechanical Force Signals

Mechanomedicine is an emerging field which aims to study the mechanical properties of cells and tissues coupled with a specific disease. In particular, in the three-dimensional tumor environment, matrix stiffness is recognized as a critical factor in cancer progression and metastatic invasion [70]. The stiffness of a specific tissue is mainly defined by the composition of the ECM and by the relative percentage of the macromolecules present in it, in particular, fibrous-forming proteins, such as collagens, elastin, fibronectin, laminins and others that build the complex three-dimensional matrix network [71]. Over the years it has become clear that the ECM can also modulate cell behavior through its physical and mechanical properties [72,73,74,75]. The tissue’s stiffness is defined by and oscillates in a specific range of Young’s modulus (a parameter indicative of the tissue elastic properties) values, spanning from the 11 Pa of intestinal mucus to the 20 GPa of cortical bone [76].

Proliferation of tumor cells leads to a desmoplastic response and to an increase in the solid stress exerted by surrounding tissues. This generates compression of tumor vessels with consequent tissue hypoxia that stimulates local inflammatory responses and cancer-associated fibroblasts (CAFs) activation [76,77]. In this activated state, CAFs overproduce ECM proteins, mainly collagen I and fibronectin, secrete cytokines and growth factors and exert contractile forces modifying tissue architecture [77,78]. In this process, the ubiquitously expressed cytokine TGF-β, which is involved in tumor cell adhesion and metastasis, is mainly used to regulate ECM production and crosslinking of collagen [79]. Moreover, radiotherapy can further increase TGF-β levels [80], which can lead to ECM deposition. It is noteworthy that, in breast cancer, one of the main results of the dysregulated matrix synthesis and CAFs activation is a reorganization of the topography of the ECM toward linearization of the ECM fibers [81], leading to an increase in breast cancer invasion [82,83].

Stiffness increase in tumors has been shown to be fundamental in the regulation of many biological effects [9,29,36,70,77,81]. In particular, it has been reported that ECM stiffness can modulate the DNA damage response to radiation due to the ability of integrins to detect mechanical stimuli. These proteins represent key mediators of cell adhesion and are able to detect changes in the microenvironment via actin and nuclear envelope proteins, making it possible to adjust the nuclear stiffness to match the microenvironment stiffness [84,85,86,87]. Cellular environments with a low stiffness lead to a soft nucleus, whereas the stiffer supports yield a stiff nucleus [88]. Deng et al. proposed that MAP4K4/6/7-mediated phosphorylation of ubiquitin leads to DSB repair deficiency in cells at low stiffness condition, which matches the proapoptotic status in soft tissues [89]. Cho et al. also reported that a high ECM stiffness promotes nuclear rupture, leading to increased DNA damage [90]. Moreover, Suzuki et al. reported a specific dynamic DDR in a model of reconstituted human skin. The authors found that DNA damage-induced foci were differently formed in different cell layers, providing a practical model for studying DNA damage response in a complex 3D environment [91]. These observations suggest that ECM could be a key extracellular regulator of DSB repair efficiency, making 2D cell models unsuitable for modeling DNA repair and, consequently, cell survival after radiotherapy. Three-dimensional models may take into account mechanical parameters and represent a more accurate model of tumor tissues on which to perform radiobiological research.

#### 3.3.2. Tumor Stroma Radioprotective Effect

The TME is composed by cancer cells and by many different non-cancerous cell types, including endothelial cells, pericytes, immune cells and fibroblasts. The pivotal components of the TME are the CAFs, which are morphologically fibroblast-like cells that originate from different tissues or precursor cells [92,93,94]. CAFs are mainly derived from normal fibroblasts that are transformed by the TME and have a role in desmoplasia induction and metabolic and immune reprogramming of the TME with an impact on adaptive resistance to radio- and chemotherapy [86]. Results obtained from research performed by irradiating normal fibroblasts or CAF are often, and erroneously, treated together. There are important differences between these cells that should be taken into account. Normal fibroblast activation is usually associated with tissue injury and wound healing, but this activation is reversible. When in the TME, fibroblast activation becomes irreversible and associated with secretory phenotypes, specialized ECM remodeling ability, robust autocrine activation and dynamic immunomodulatory signaling functions, since TME provides continuous and persistent injurious stimuli, leading to the persistent state of activation of CAFs [94,95]. Irradiation of normal fibroblasts or CAFs present in the TME induces different effects [95] that should be considered in radiobiological research.

Among non-cancerous cells, endothelial cells are possibly the beststudied components of TME, and it is well known that radiation induces endothelial cell dysfunction, including apoptosis, increased permeability and detachment from the basement membrane [96,97]. Endothelial dysfunctions lead to several effects, including increased extravasation and subsequent metastasis [97,98].

Cancer cells also interact with a plethora of other cells, specific to different tumors, whose biological response to radiation can be significantly modulated by TME. For example, in the brain microenvironment, tumor cells interact with astrocytes, pericytes, oligodendrocytes and neurons, while in the breast tumor microenvironment, tumor cells mainly interact, among others, with fibroblasts, adipocytes and myoepithelial cells. It then becomes clear that TME alterations inevitably lead to repercussions for tumor progression and the possibility of recurrence [9,99,100,101].

In the TME, cell–matrix interactions are mainly mediated by integrins [102,103]. The role of integrins in modulating radiotherapy effects is described in many works. It has been reported that radiotherapy (18 Gy) increased the expression of integrins α2, β1 and α5 and dramatically augmented and redistributed focal contacts in pancreatic cancer [104]. β1 integrin is known to modulate the cellular response to radiation by stimulating cell proliferation of pancreatic cancer cells [105].

In conclusion, it is crucial to consider the entire TME and not only the single cells when studying the effect of radiotherapy on a tumor, since the complex and coordinated radiation response of a tumor cannot be effectively reproduced in an extremely simplified system such as that of in vitro cultures lacking both the three-dimensionality and the complex interactions between cells of different types and the extracellular matrix.

## 4. Three-Dimensional Cellular Models for Radiobiological Studies: A Look into the Future

**Figure 2:**
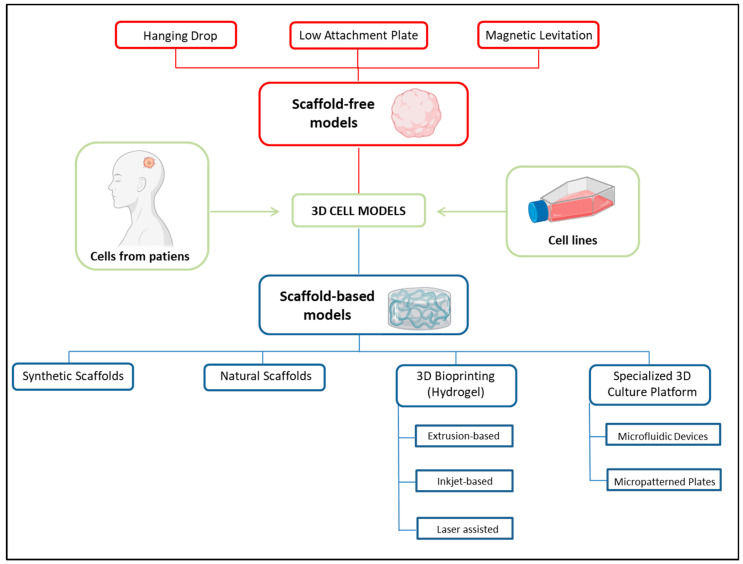
Different approaches for 3D cell culture model development. Cells included in the 3D model can derive from isolated primary cells or from cell lines. The microenvironment can be mainly represented by either scaffold-free or scaffold-based strategies. The scaffold-free approach relies on the self-aggregation of cells in specialized culture plates, such as hanging drop microplates or low adhesion plates with low attachment coating, or in the presence of a magnetic field. The scaffold-based approach is instead based on the use of a scaffold that provides physical support to the cells and encourages cell growth and differentiation. The scaffold can be made by synthetic or natural biopolymers, by hydrogels (3D bioprinting) or by polymeric hard platforms that can include microfluidic networks. Created with BioRender.com and adapted from free BioRender templates (2020).

Three-dimensional systems have gained in popularity in recent years due to their significant advantages in mimicking human tissues and overcoming the limitations of 2D cell culture systems. Several models have been proposed in order to optimize the ECM composition and cell interactions, modeling tissues with higher fidelity and providing more suitable platforms to be used in drug testing and cancer treatment response [28,106]. A complete description of the various 3D models available is beyond the scope of this paper and the reader should refer to the literature for further details [10,107,108]. In brief, 3D cell models can be basically categorized into scaffold-free and scaffold-based models (Figure 2): the first group includes models that do not require external structural supports, while the second group includes models in which cells are seeded and grown on 3D structures (scaffolds) made of synthetic, natural or mixed components that provide physical support [11,108]. In the latter case, the scaffold properties, such as permeability, stiffness, surface chemistry and adhesive moieties, are essential in mimicking the cell’s microenvironment. Specific biologically active molecules, such as growth factors and hormones, can be encapsulated inside some types of scaffolds to improve and stimulate cell growth and proliferation [109,110].

Each of these models has strengths and weaknesses, so that the researcher should evaluate the model that best suits their needs in order to obtain the best results.

The newer 3D cell models have been widely explored in the past decade, but few of them have been used in radiobiological studies. In the following paragraphs, some of the most recent and innovative 3D models will be described, in order to show their potential in the radiation field and stimulate their use in radiobiological research.

### 4.1. Organoids

Organoids are complex 3D models that comprise multiple cell types originating from tissue-specific adult stem cells, embryonic stem cells or induced pluripotent stem cells by self-organization, providing platforms for drug screening and cancer research [111]. Despite the limitations of organoid cultures, including lack of interactions with the immune system, patient-derived organoids show important advantages such as maintenance of chemoresistance and genetic mutations that commonly appear in original tissues [112]. Organoids have been established following different protocols to mimic many organs [113], but it is only recently that organoids have been used in radiobiological studies.

One of the most studied and well-established organoid models is the gastrointestinal “mini-gut” organoid, a 3D model originally established from mouse small intestinal stem cells [114] and subsequently from humans from various different locations along the gastrointestinal tract [115,116,117]. Although these models have opened novel avenues of study for intestinal development, there are limited studies using organoids to investigate the effect of radiation on the gastrointestinal tract. In several works, organoids have been used alongside in vivo studies to determine the effect of radioprotective or radiosensitising molecules in gastrointestinal normal and tumor tissues [118,119]. Other works compared the effects of radiation on 2D and 3D models, showing the higher radioresistance in the latter. A very interesting work by Vincent-Chong and Seshadri recently demonstrated that the radiation dose required to achieve 50% of cell death in 2D culture and 3D organoids obtained from a murine oral squamous cell carcinoma was 2.4 Gy and 12.6 Gy, respectively, highlighting the higher radioresistance of cells grown in a 3D microenvironment [120]. Moreover, another work reported highly similar dose–survival curves of organoids derived from small and large intestine crypts and an in vivo mouse model, defining conditions for delivery of radiation to intestinal organoids that would accurately reflect the impact of ionizing radiation on the organ of origin [121]. Park et al. conducted a co-clinical trial to analyze the correlation between the irradiation response of individual patient-derived rectal cancer organoids and the results of actual radiotherapy through a machine-learning-based prediction model. The authors showed that the radiation response of organoids could predict the patient’s tumor regression grade with statistical significance [122].

Brain organoids have also been established and characterized. Among brain tumors, GBM represents a highly aggressive brain tumor with an extremely poor prognosis [123]. Recently, new organoid models have been established that may help to understand the bases of GBM radioresistance [124,125], considering the effect of the TME and overcoming limitations of the more traditional cell models. Indeed, organoids can recapitulate limitations in oxygen and nutrient availability, resulting in gradients that stimulate GBM self-renewal and promote maintenance of a stem-like cell state [126,127], giving rise to a heterogeneous cell population. This is fundamental to mimicking the GBM radiation response, since, while the non-stem cells of the organoids were radiosensitive, the tumor-initiating cancer stem cells were resistant [124].

Verduin et al. recently reviewed studies using cancer organoids to identify new anti-cancer treatments, discussing the limitations and improvements needed to obtain a more realistic model for use in chemoradiobiological studies [128].

In general, organoids provide more favorable conditions than traditional cell line models for tissue growth and structural organization, but they do not retain the complexity of a real tissue due to the lack of TME. To overcome this limitation, organoids co-cultured with TME (i.e., CAF, T lymphocytes) components have recently been introduced [129,130], providing more reliable results.

One of the main limitations of organoids is that, while they are capable of simulating the structures and functions of organs in vitro, they have a limited ability to develop a complex vascular network that accurately recreates the interaction between tissues and vascular systems. As a result, organoids often struggle to sustain themselves due to insufficient oxygen and nutrient supply, as well as the accumulation of metabolic waste. Despite numerous attempts to incorporate functional vasculature into organoid models, the establishment of vasculature within organoids that connects with an external perfusable vascular network has only been achieved through transplantation into host animals (as reviewed in [131]).

### 4.2. Organ-on-a-Chip Microfluidic Culture Devices

Organ-on-a-chip microfluidic (OCM) culture devices represent one of the newest in vitro systems able to recapitulate organ-level and even organism-level functions. In general, microfluidic platforms are based on the presence of hollow channels forming a network in which fluids can circulate under a laminar flow. This approach allows us to simulate interactions between organs by fluidically coupling two or more miniature tissues grown and residing in the microfluidic chips [132]. The first devices were designed to work with 2D cell cultures, but due to the sudden technological advances in 3D systems, biomaterials, bio-manufacturing and microsystems technology, new devices were engineered to work with 3D cell cultures, organoids and bioprinted tissues. The goal is to obtain tailored chips that, by trying to recapitulate the physiological and physical characteristics of specific tissues, may allow the study of potential interactions of one organ with at least one other through soluble signaling molecule exchange. Two approaches can be used in the OCM design: (a) engineered tissues, organoids or tissues from biopsies can be incorporated into the chip and connected; (b) primary or immortalized cells, or stem-cell-derived sources can be grown inside a device designed to support the remodeling of cells into a functional tissue [133]. Moreover, two main device architectures can be used: (a) solid organ chips, which include 3D tissue masses (i.e., liver, tumor, adipose tissue) [134,135,136]; (b) barrier tissue chips, in which cells are arranged to form a natural barrier between fluid compartments [137,138].

The first microfluidic culture device was engineered twelve years ago to recapitulate the lung alveolus, and was formed by two parallel hollow channels mimicking the air–liquid interface [137]. Since then, many organ-on-a-chip devices have been developed and used to model the pathophysiological processes underlying a wide range of diseases and to study the response of one or more organs to drugs or to various insults [132]. One of the main advantages of this kind of model system is that working on the microscale offers the possibility to finely control the microenvironment, which is carefully engineered inside the devise [133,139,140]. Moreover, being a microengineered device, the chip can also integrate various types of in-line sensors able to monitor parameters such as oxygen levels, tissue viability or electrical activity [141,142,143]. Three-dimensional microfluidic chips can take various forms and architectures, and can be built using different materials such as glass, polydimethylsiloxane, poly(methyl methacrylate), polycarbonate, polystyrene or polyurethane depending on the system to be recreated, and on biocompatibility and manufacturing strategies [144]. Moreover, many devices are designed with optically clear materials that enable real-time high-resolution microscopic imaging even if the presence of thick tissues can make the analysis more difficult. It should be considered that some kinds of analysis, such as transcriptomics, proteomics or histological analysis, could require the chip to be dismantled, and this represents a drawback since it could interfere with the three-dimensional structure of the tissue [145].

One of the main limitations of these systems is the difficulty of standardization, and it is still fundamental that results obtained from the OCM are systematically validated with those obtained using conventional in vitro and in vivo models to make sure that OCM results are due to real alteration in the biological response to stimuli and not a result of experimental artifacts [133]. OCMs can also be very difficult to handle, since they include external pumps, tubing, connectors, and a valve to operate, and the connection of devices to the pump requires the manual ability of expert researchers [133]. The use of OCM in radiobiological studies is challenging, and some considerations have to be made when performing an experiment involving irradiation. In particular, it is imperative to consider whether the type of radiation used can penetrate through the materials used to construct the OCM. An accurate dosimetric evaluation has to be performed in order to avoid the effects of the degradation of the radiation along the microdevice biomaterials.

Very few works using OCM have been reported. Jalili-Firoozinezhad et al. reported that the human gut chip may serve as an in vitro platform for studying radiation-induced cell death and associated gastrointestinal acute syndrome by using 8 Gy dose of γ-rays [146]. Cheah et al. employed a bespoke microfluidic device to maintain a head and neck squamous cell carcinoma tissue whilst subjecting it to external x-ray beam irradiation. They also measured the tissue’s response using a panel of cell death and proliferation markers. While the authors acknowledge limitations in maintaining the viable biopsy in the device, their study shows the potential of the microfluidic-irradiation model to determine the response of an individual’s tumor to irradiation [147]. In another study, Patra et al. developed a chip to study the effects of photon irradiation on sarcoma-derived spheroids to evaluate the combined effect of radio and chemotherapy [148]. To date, to the best of this author’s knowledge, no studies have been performed to evaluate the radiation-induced modulation of the crosstalk between immune system and cancer TME or the radiation-induced bystander effect on a chip.

### 4.3. Three-Dimensional Bioprinting

**Figure 3:**
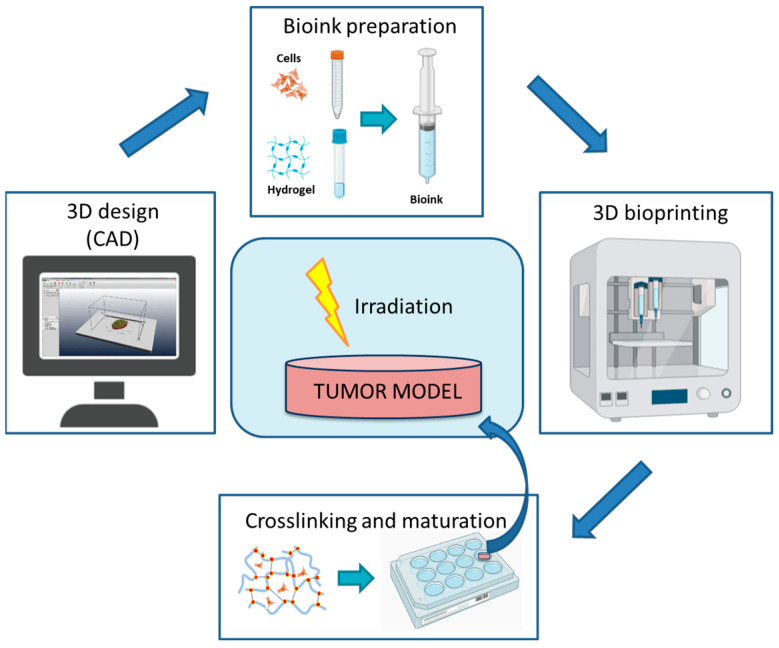
Main steps in tumor model development using 3D bioprinting. Created with BioRender.com and adapted from free BioRender templates (2020).

Among the approaches used to develop 3D cell models, 3D bioprinting is the most futuristic. Three-dimensional bioprinting utilizes computer-controlled systems to automatically deposit biological materials, biochemicals and living cells layer-by-layer to fabricate complex user-defined 3D objects [149,150,151]. Using this technology, it is possible to print cells and cell aggregates encapsulated into hydrogels [152]—water swollen networks of polymers—mimicking pivotal elements of native extracellular matrices. The first phase of the 3D printing process consists of the elaboration of the model to be printed using a computer-aided design (CAD) program. In the second phase, the model is printed using a bioink, a liquid mixture of cells with biocompatible hydrogel. The hydrogel-based biomaterials used in 3D bioprinting must possess adequate viscoelastic properties to allow a correct ink deposition [153]. The 3D bioprinting process produces well-defined structures in all three dimensions, with high resolution and reproducibility. After the bioprinting process, a post-printing phase is necessary to harden the bioink and create a stable structure through an appropriate crosslinking approach [154]. Finally, the construct needs a maturation phase in the incubator to allow cells to grow and interact (Figure 3).

It is of fundamental importance that the printing process, the hydrogels used and the post-printing processes are able to support good cell viability and allow tissues to develop functionality after printing [155]. The entire process of 3D bioprinting aims to produce physiologically relevant models able to reproduce, in the best possible way, the architecture of a real tissue, the interactions between cells and between cells and the microenvironment, the metabolic pathways and the biological characteristics of in vivo tissues.

Hydrogels have demonstrated their utility in various cell culture applications, uncovering fundamental processes that govern cell behavior and offering novel techniques for the growth and controlled differentiation of different cell types, surpassing the capabilities of traditional culture surfaces. When choosing a hydrogel, several factors should be taken into account. For biologists, the most crucial considerations include the gel’s cell adhesivity and whether it occurs naturally or through modifications, its stability in a culture environment and its biophysical characteristics, such as the elastic modulus of the hydrogel [156]. The selection of bioinks depends on various factors, including the printing method employed, the specific types of cells utilized, and the mechanical, physical and chemical requirements of the system [152]. Each type of hydrogel has specific features, advantages and disadvantages (summarized in Table 1), and its use must be evaluated on the basis of various factors, including the printing conditions, the cells used and the results to be obtained.

Among the main advantages of the 3D bioprinted models on the other 3D models is the possibility to control the extracellular matrix stiffness [157].

Along with the development of increasingly effective technologies for recreating in vitro tissues using 3D bioprinting, various models of both healthy and tumor tissues have been developed [158,159,160]. The major limitation of the other tumor models lies in replicating exact tumor physiological conditions, including the TME composition in terms of matrix and cells composition. The engineering of tissue-like constructs as tumor models with similar cellular and ECM compositions could really improve the predictive value of such models. Indeed, the possibility to recreate the TME in a controlled way is intriguing. For example, 3D bioprinting allows us to print both malignant and nonmalignant cells to recreate a TME where secretion of factors such as cytokines and matrix remodeling enzymes can contribute to the tumorigenic process [161,162,163]. A very interesting and complex GBM model has been developed by Tang and coworkers in which patient-derived GBM stem-cells, macrophages, astrocytes and neural stem cells were bioprinted to create a biomimetic 3D cancer microenvironment [164]. This model represents an example of how 3D bioprinting technology can lead to the creation of a customized model in which the TME is highly similar to the real one. Of course, the use of such a model in the field of personalized medicine could be incredibly useful. Mondal et al. developed a hydrogel to print non-small cell lung cancer patient-derived xenograft cells and lung CAFs co-cultures, showing that this model can be used for studying high-throughput drug screening and for other pre-clinical applications [165]. Langer et al. used multicellular scaffold-free tumor tissues incorporating multiple cell types of breast and pancreatic tumors, including patient-derived cells, showing the ability of cells to self-organize, secrete extracellular matrix factors and respond to extrinsic signals [166].

Many other works have reported the possibility of recreating the main characteristics of tissues and tumors, including vascularization [22,167,168,169,170,171,172]. Different approaches have been developed to implement the 3D model with blood vessels or endothelial components. Yi et al. successfully developed an ex vivo cancer model that closely mimics the complex ecosystem of the glioblastoma, incorporating essential cues to recapitulate its pathological characteristics, including the presence of endothelial cells that form the blood–brain barrier [22]. A different approach has been reported by Han et al., who describe a bioprinting technique that enables the recreation of the TME while maintaining control over spheroid size. The TME was constructed by printing a layer of blood vessels composed of fibroblasts and endothelial cells within a mixture of gelatin, alginate and fibrinogen. Subsequently, multicellular tumor spheroids derived from glioblastoma cells were seeded onto the blood vessel layer. The researchers observed that the presence of multicellular tumor spheroids led to the generation of sprouts from the blood vessels, resulting in an increased spheroid size within the surrounding environment [173]. A different strategy involves the direct printing of vascular scaffolds. Kolensky et al. reported a method for bioprinting 3D tissues containing cells and vasculature that exceed a thickness of 1 cm. These tissues could be perfused on a chip for extended periods exceeding 6 weeks. Their approach involved the integration of multiple cell types, including human mesenchymal stem cells and human neonatal dermal fibroblasts, within a customized extracellular matrix ink. Additionally, embedded vasculature was incorporated into the tissue, which was then lined with human umbilical vein endothelial cells. By co-printing these various inks, researchers successfully created a single, thick tissue consisting of parenchyma, stroma and endothelium, enabling long-term perfusion on a chip [174].

While several important results have already been reported to assess tissues and tumors drug sensitivity, to the best of this author’s knowledge, only a few 3D bioprinted models have been used for radiobiological studies [22,175]. Yi et al. reported a patient-specific 3D-bioprinted GBM model consisting of a core region containing the patient cancer cells surrounded by vascular endothelial cells mimicking the blood–brain barrier [22]. This model was used to evaluate the resistance of patients to chemoradiotherapic treatment. The authors showed that the bioprinted model was able to reproduce clinically observed patient-specific resistances to treatments. Al-Zeer et al. recently reported the results of a pilot study to evaluate the suitability of 3D-bioprinted samples for experimental radiotherapy, showing how 3D structures generated from human lung cancer cells with 3D bioprinting can be used to study the effects of radiotherapy in a standardized manner [175].

**Table 1:** Summary of representative hydrogels that can be used for 3D bioprinting with their main advantages, disadvantages and features.

Material	Advantages	Disadvantages	Material Features	References
Collagen	Enhanced cellular attachment and growth Biodegradable Biocompatible	Gelation occurs at high temperatures, while it remains liquid at lower temperatures	Routinely obtained from the tendons of rat tails or the skin/tendons of cows; commonly available in pepsin- or acid-solubilized form; susceptible to enzymatic degradation; it possesses structural and mechanical characteristics similar to natural tissues and provides native cell adhesion ligands.	[156,176,177]
Alginate	Rapid ionic crosslinking Biocompatibility Cheap to produce The strength of the hydrogel can be adjusted by modifying the percentages of monomers used, allowing for tunability.	Biologically inert Limited biodegradability Poor stability Low mechanical and barrier properties	Polysaccharides composed of β-D-mannuronic (M) and α-L-guluronic (G) acid units. Structural organization depends on alginates’ natural sources.	[178,179]
Fibrin	Enzymatic crosslinking High cell adhesion, growth and development Ability to carry multiple cells and therapeutic factors Natural degradation	Mechanical Instability Rapid degradation High post-crosslinking viscosity May cause immune reactions	Fibrin is derived from the crosslink of fibrinogen present in the blood; viscoelastic polymer that possesses both elastic and viscous properties; provides a good substrate for studying the wound healing processes in vitro.	[174,176,177,180,181]
Hyaluronic acid	High biocompatibility Excellent hydrophilicity Reproducibility	Poor mechanical strength and fast when used pure.	Non-immunogenic natural polymer present in the extracellular matrix of various tissues; it can be chemically modified and mixed with a printable hydrogel to form a HA-based hydrogel solution.	[182,183]
Polypeptides	Possibility of developing customized peptide sequences Good interactions with cells Good degradability	High cost Achieving long-lasting gel formation with mechanical properties suitable for strong cell traction can be challenging	Peptide self-assembly in nanostructures; synthetic materials with tunable properties.	[156,184,185]
Commercially available hydrogel	Biodegradability, biocompatibility, low immunogenicity and ease of usage	High cost	Sold as combinations of different natural and/or synthetic components, to obtain specific hydrogels for the growth of specific cell types.	[186,187,188]

The potential of 3D bioprinting in the field of radiotherapy is therefore still to be investigated, as it potentially represents the most suitable technique for the development of in vitro 3D models with the best ability to mimic in vivo tissues and tumors.

## 5. Discussion and Conclusions

The linear–quadratic model is a key model used in radiobiology and physics to provide a simple relationship between cell survival and delivered dose, and is useful for defining the response of healthy and tumor cells to different doses of radiation. However, despite the LQ model’s ubiquity, there are still questions to be answered, including whether an in vitro single cell survival model can truly represent clinical tissue response. Several works have reported that tumor models have shown increased radioresistance in the presence of other cells of the TME and/or the ECM components [28,104]. Indeed, it seems clear that the mechanisms of response to radiation are largely modulated by the extracellular environment which can directly or indirectly control gene expression, DNA repair, cell morphology, proliferation and many other processes. In this scenario, 3D cell systems, in which different cells can cohabit in the same microenvironment, interacting with each other and with the extracellular matrix, represent a key tool for understanding the effects of radiation on biomimetic tissues in a way much more representative of reality. The different 3D cell models currently available for research should be carefully evaluated and used to redefine dose–response curves in a finer and more representative way, allowing a more precise definition of treatment plans.

**Figure 4:**
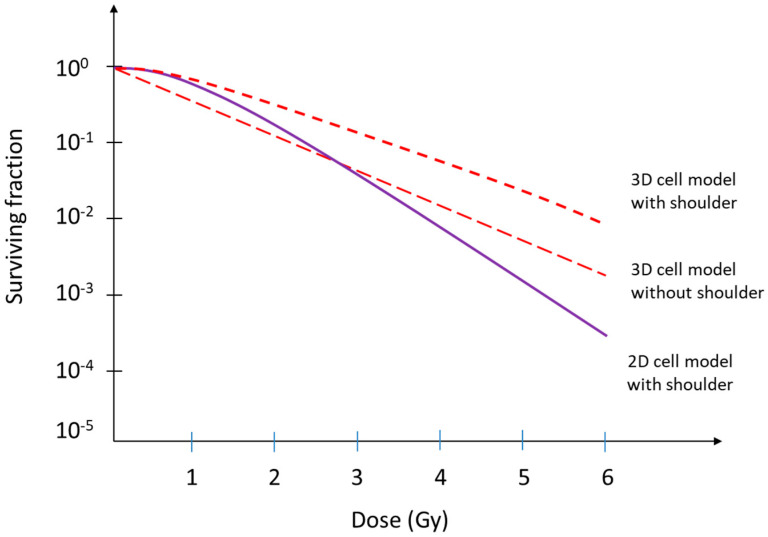
Shape of survival curve for mammalian cells exposed to radiation. The violet curve represents a generic dose–response curve [27], while the red dotted lines represent two hypothetical curves that could be obtained by irradiating cells grown in three-dimensional systems capable of more accurately mimicking human tissue and that, in such an environment, appear to show greater radioresistance [10,24,33,34,106].

Survival curves for mammalian cells are usually presented in the form of the violet curve shown in Figure 4, with dose plotted on a linear scale and surviving fraction on a logarithmic scale. Qualitatively, the shape of the survival curve can be described, for low-LET radiations, as a curve starting out straight on the log-linear plot with a finite initial slope, bending and straightening again as the dose increases. The curve is described by several parameters of curve slope and by the width of the shoulder, representative of the cell’s ability to repair radioinduced DNA damage at low doses. For high-LET radiations, the cell survival curve is a straight line drawn from the origin [27]. Historically, these curves have been determined using conventional two-dimensional cell cultures, which, after being irradiated, have been analyzed for the ability of surviving cells to create cell colonies [6,27].

However, if the first observations on 3D cell cultures reporting a much higher radioresistance than observed in 2D cultures [15,24,33,34,67,68] can be confirmed, the radiation effects on tissues and tumors could be different in reality than what has been reported so far. The red curves depicted in Figure 4 represent hypothetical radiation response curves that, taking into account the radioprotective role of the microenvironment, may be more representative of reality.

It is noteworthy that the limited number of studies reported in the literature have presented dose–response curves that highlight the differences in radiation response between 2D and 3D models. Interestingly, these initial findings consistently indicate increased radioresistance of cells cultivated in a 3D environment compared to those grown in a 2D setting [15,34,35,189]. For example, Raitanen et al. conducted a recent study focusing on spheroids derived from various human cancer cell lines. The study findings indicate that the radiobiological response observed in 2D cultures does not accurately represent the response observed in 3D cultures when exposed to X-rays [24].

Table 2 provides an overview of the key studies conducted in the field of radiobiology using the 3D models described in this review, with a specific focus on those studies that report dose–response curves.

**Figure 5:**
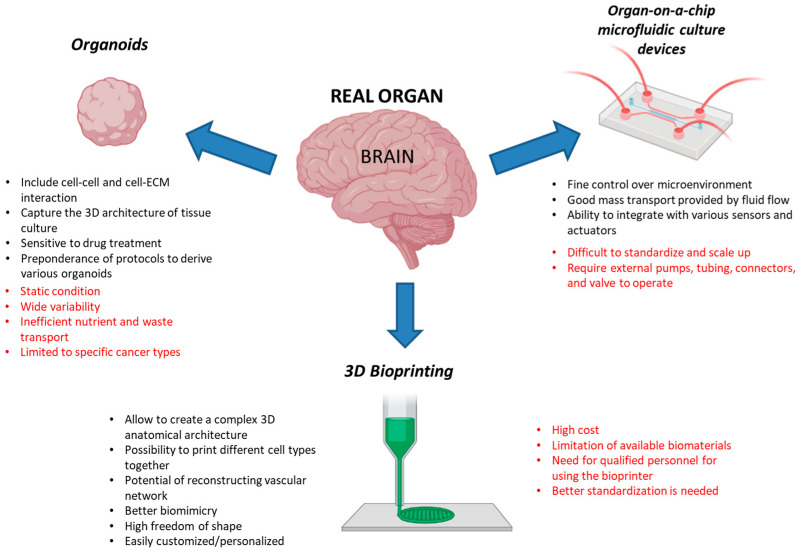
Advantages (black) and disadvantages (red) of three major and latest 3D cell systems. Created with BioRender.com and adapted from free BioRender templates (2020).

A noteworthy alteration in the α/β ratio holds direct clinical relevance when it comes to choosing optimal fractionation schedules in the field of radiation oncology. This change directly affects factors such as the dose per fraction, dose fractionation, and dose rate in combined treatments. There is therefore an urgent need to understand the differences in cell response to radiation between 2D and 3D cell models. To this aim, several 3D models are now available with different advantages and disadvantages (Figure 5). For example, while there is a preponderance of protocols to derive various organoids that can be modified by different laboratories depending on their capabilities, 3D bioprinting has the unique capacity to recreate a complex and defined microenvironment, including tissue structure and stiffness. At the same time, while a wide variability has been noted in organoids cultured under identical conditions [190], 3D bioprinting stands out as a technology that is difficult for many laboratories to afford today.

Moreover, there is often a difficulty in effectively extrapolating findings from these model systems to human tumors, particularly when it comes to accurately predicting drug or radiation sensitivities. To address this challenge, a variety of machine learning approaches have been tried. For example, Price et al. introduced a methodology that employs joint dimension reduction (jDR) to horizontally merge gene expression data across various model systems and human tumor groups. They subsequently applied this technique to merge gene expression data from human cancer cell lines and mouse model tumors with the human repositories of high-dimensional multi-omics (The Cancer Genome Atlas (TCGA)) [191]. Mourragui et al. developed a computational framework that constructs a consensus space, capturing shared biological processes between preclinical models and human tumors. Leveraging this space, the researchers developed drug response predictors that effectively transfer from preclinical models to human tumors, ensuring robustness and reliability [192]. Gomez-Roman et al. employed a different approach, utilizing a customized 3D cell culture system that mimics essential histological characteristics of the glioblastoma. Through this system, they successfully replicated the clinical outcomes of three molecular targeted therapies. This demonstrated the reliability of the 3D model in predicting clinical efficacy, highlighting its superiority over conventional 2D models, which have historically failed to accurately predict clinical outcomes [193].

It is clear that the potential of 3D models is manifold, and it is necessary for the scientific world to move in this direction. While, at the moment, radiobiology remains stuck on the data obtained over decades from 2D cell models, it would be fundamental to evolve to using more representative 3D cell models in vitro in order to improve radiation therapy plans by making them more efficient. Despite this, the transition from 2D to 3D cell cultures for radiobilogical studies is associated with some concerns. The first issue is the limited utilization of 3D models in radiobiology which reflects a limitation in the availability of sufficient data and studies using these models and a poor understanding of how findings from 3D models can be extrapolated and applied to real human scenarios. Despite offering several advantages compared to 2D cultures, 3D cultures tend to be more expensive and can pose challenges in replicating cell microenvironments, particularly when utilizing certain 3D culture techniques [10]. When large scaffolds are utilized, imaging can become challenging, since fluorescence microscopy, commonly used in 2D cell cultures, presents difficulties in 3D cell cultures. With the increasing complexity of 3D cell models, there is a need for more sophisticated tools to analyze them. However, the majority of analysis techniques currently in use were originally developed for 2D cell cultures, making the transition to 3D less than ideal [194].

An important challenge to be considered in the future is the standardization of 3D models and radiation biology protocols, so that consistent and reproducible results can be obtained with the various 3D models used. The absence of a standardized method for 3D cultures is a noteworthy concern, as it poses challenges in establishing a consistent technique across different cell types [195,196]. However, this very flexibility and adaptability are what contribute to the ongoing progress and advancement of 3D systems. Furthermore, the establishment of 3D cultures is a demanding task due to the intricacy and precision involved in identifying suitable cell types and scaffold materials that facilitate the development of an adequate ECM and vascularization. In the context of cell culture models, the presence or absence of an ECM scaffold is a fundamental distinction between 2D and 3D cultures. However, the specific impact of ECM composition on cells cultured in a 3D environment has not been uniformly established or defined. The variability in ECM composition can introduce uncertainty and potentially influence the outcomes of drugs and radiobiological studies conducted using 3D cell cultures. Consequently, it is crucial to conduct research aimed at studying and comprehending the role of ECM in 3D cell culture systems. Despite these limitations, as more researchers transition to 3D cell culture, the development of new methodologies to overcome the existing limitations will be accelerated. With increased adoption of 3D models, there will be a growing focus on finding innovative solutions to address the challenges associated with 3D cell culture. 

## Figures and Tables

**Table 2:** Description of reported 3D models applications in radiobiology. Studies reporting the comparison of dose–response curves of 2D vs. 3D models are highlighted in bold in the references.

3D Model	Cells	Structures	Description of Application	References
**Organoids**	Cells from human colon tissues	Human nonmalignant colon organoids	Study demonstrating that auranofin pretreatment can prevent radiation toxicity and improve cell survival.	[118]
**Organoids**	RP-MOC1 cells from a mouse tongue tumor	Tumor tongue organoids	Evaluation of the radiation response in different cellular models of tongue tumor.	**[120]**
**Organoids**	Mouse intestinal stem cells	Intestinal organoids	Generation of an in vitro radiation sensitivity assay validated against published data using classic in vivo radiobiology concepts.	**[121]**
**Organoids**	Cells from human rectal cancer biopsies	Rectal cancer organoids	Correlation between the irradiation response of individual patient-derived rectal cancer organoids and the results of actual radiotherapy through a machine learning-based pre-diction model.	**[122]**
**Organ-on-a-chip**	Human endothelial cells (HUVEC) Human colorectal carcinoma cell (Caco-2)	Microfluidic Gut-on-a-Chip device	Modeling radiation injury-induced cell death and countermeasure drug responses.	[146]
**Organ-on-a-chip**	Cells from human head and neck squamous cell carcinoma (HNSCC)	Devices with microfluidic-perfused HNSCC biopsies	Investigation on the response of head and neck squamous cell carcinoma (HNSCC) tissue to irradiation using a microfluidic device.	[147]
**Organ-on-a-chip**	Primary human soft-tissue sarcomas (STS) cell lines	Microfluidic platform containing STS spheroids	Proof-of-concept experiments to determine if this device could be used for the screening of radiosensitizing and radioprotective agents.	**[148]**
**3D bioprinting**	Human glioblastoma (U-87) and endothelial (HUVEC) cell lines	Glioblastoma model surrounded by the blood brain barrier	Development of patient-specific ex vivo models of glioblastoma tumors using bioprinting technology, able to replicate the pathological characteristics and complex ecology of native tumors, providing a tool for determining personalized cancer treatments for individual patients.	[22]
**3D bioprinting**	A549 cell lung cancer cell line	3D bioprinted constructs	Pilot project to evaluate the suitability of standardized samples generated from 3D printed human lung cancer cells in radiotherapy studies.	[175]

## Data Availability

Not applicable.
